# Investigation of Atyp.C using UF-5000 flow cytometer in patients with a suspected diagnosis of urothelial carcinoma: a single-center study

**DOI:** 10.1186/s13000-020-00993-1

**Published:** 2020-06-25

**Authors:** Chunyun Ren, Xing Wang, Chunwei Yang, Shengchao Li, Shuangchun Liu, Hongcui Cao

**Affiliations:** 1 Department of Laboratory Medicine, The First Affiliated Hospital, College of Medicine, Zhejiang University, 79 Qingchun Rd, Hangzhou City, 310003 China; 2 Key Laboratory of Clinical In Vitro Diagnostic Techniques of Zhejiang Province, 79 Qingchun Rd, Hangzhou City, 310003 China; 3 Department of Pathology, The First Affiliated Hospital, College of Medicine, Zhejiang University, 79 Qingchun Rd, Hangzhou City, 310003 China; 4 State Key Laboratory for the Diagnosis and Treatment of Infectious Diseases, The First Affiliated Hospital, College of Medicine, Zhejiang University, 79 Qingchun Rd, Hangzhou City, 310003 China; 5 Zhejiang Provincial Key Laboratory for Diagnosis and Treatment of Aging and Physic-chemical Injury Diseases, 79 Qingchun Rd, Hangzhou City, 310003 China

**Keywords:** Atyp.C parameter, UF-5000; urothelial carcinoma, Urinalysis

## Abstract

**Background:**

This study evaluated the predictive power of Atyp.C (a parameter of UF-5000 flow cytometer) for patients with a suspected diagnosis of urothelial carcinoma.

**Methods:**

We analyzed 163 urine specimens from 128 patients with suspected urothelial carcinoma using a fully automated fluorescence flow cytometry analyzer (UF-5000) and evaluated its performance on identifying atypical/malignant urothelial cells. From January 1, 2019 to April 4, 2019, all consecutive specimens for urinary cytopathology were enrolled.

**Results:**

Of the specimens with urinary cytopathology, 67 specimens (41.1%) revealed abnormal findings in cytology analysis. Among them, 20 specimens (12.3%) were diagnosed as atypical urothelial cells, 26 specimens (16.0%) as suspicious for malignancy (S-malignancy), and 21 specimens (12.9%) as confirmed malignancy. The UF-5000 findings were positive in 59 specimens (36.2%); therefore, the agreement with cytopathology was 73.0%. Using follow-up histologic diagnosis of urothelial carcinoma with or without urinary tract cytology (UTCy) as a reference standard (suspicious and confirmed malignancy were the positive criteria for UTCy), the sensitivity was 59.0%, specificity was 82.1%, positive predictive value was 75.0%, negative predictive value was 68.8%, and the agreement was 71.1%.

**Conclusions:**

It is worth knowing and reporting that the Atyp.C assay may be used as an accessory test for patients with suspected urothelial carcinoma, based on its ability to identify high-risk patients who might need closer follow-up or additional medical treatment.

## Background

Urinalysis is a common test in clinical laboratories. Urinary sediment examination can be used to diagnose kidney and urinary tract diseases and other maladies [1–3]. Bladder cancer, which is common in men, is the 10th most common form of cancer worldwide, with nearly 550,000 new diagnoses and 200,000 deaths in 2018 [4]. Overall, 95% of bladder cancer diagnoses are urothelial carcinoma [5]. The finding of atypical/malignant urothelial cells is important for proper treatment and follow-up. Unfortunately, urinary cytology is performed in the pathology department and most laboratory medicine departments separate the routine urinalysis from urinary cytopathology. Some researchers have shown that atypical/malignant urothelial cells can be identified in routine urinalysis with the conventional urinary sediment examination or automated urinalysis [6, 7]. Urothelial cells, either atypical or malignant, should be noted and are worth reporting in routine urinalysis specimens.

The UF-5000 is a fully automated analyzer that uses fluorescence flow cytometry technology, with a new blue semi-conductor laser at 488 nm wavelength. The UF-5000 can rapidly count, recognize, and classify urine particles using forward scatter light, side scatter light, side fluorescent light, and depolarized side scattered light, in two chambers: the surface and core chambers. The UF-5000 provides a new parameter, “Atyp.C,” which uses the side fluorescence signal waveform area to differentiate between atypical and non-atypical cells, based on differences in the fluorescent staining of nucleic acids in urothelial cells. Based on the scattergram, Atyp.C parameter is able to report the number of atypical/malignant urothelial cells with abnormally high levels of nucleic acid contents. This provides important clues for a suspected diagnosis or the surveillance of urothelial carcinoma patients. Routine urinalysis can be used to identify atypical/malignant urothelial cells using automated digital or manual microscopy, but no studies have investigated the ability of the UF-5000 to detect atypical/malignant urothelial cells [6, 7]. Therefore, this study evaluated the performance of a new parameter, Atyp.C, in patients with a suspected diagnosis of urothelial carcinoma on urinary tract cytology (UTCy).

## Materials and methods

### Ethics approval

This study was approved by the Research Ethics Committee of The First Affiliated Hospital, School of Medicine, Zhejiang University (Reference No. 2018-1069). Informed consent was obtained from most hospitalized patients before enrollment. Other hospitalized patients and all outpatients were informed of their right to refuse enrollment via telephone. All procedures met the requirements of the Informed Consent Guideline.

This was a prospective study of the use of Atyp.C, determined by UF-5000, for assessment of patients with suspected urothelial carcinoma, which were ultimately diagnosed using the same UTCy specimens at The First Affiliated Hospital of Zhejiang University a few days later. All eligible patients whose UTCy specimens had been sent to the clinical laboratory for UF-5000 analysis, from January 1, 2019 to April 4, 2019, were included.

### Reference standard

Using follow-up histologic diagnosis of urothelial carcinoma with or without UTCy as a gold reference standard, we evaluated the performance of UF-5000 with Atyp.C assay. In the absence of histologic diagnosis, positive UTCy results (i.e., suspected or confirmed malignancy findings) were used as a reference standard to evaluate the predictive power of Atyp.C assay. In addition, patients with negative initial cytology but positive follow-up cytology with no available histologic follow-up were also defined as positive for urothelial carcinoma.

### Collection and preparation of urine specimens

If patients declined the ancillary test, their specimens were not sent to the clinical laboratory. To avoid erratic results for UF-5000, aggregates or visible particle-containing specimens that cannot be resuspended must be excluded. In total, 163 consecutive specimens were collected in sterile containers, including voided urine samples, bladder washings, and catheterized urine samples. No specimen in our study had to be excluded for this reason. Each specimen was divided into two sterile tubes: one for urinary cytopathology and the other for UF-5000 analysis. When samples were sent to the pathology laboratory, a ThinPrep T3000 processor (Hologic, Marlborough, MA, USA) was used to make a single slide and stained with Papanicolaou stain. Urine specimens were prepared within 2 h after voiding and analyzed by expert staff at the hospital.

### UF-5000 analysis and Atyp.C assay

The UF-5000 analyzer (Sysmex Corporation, Kobe, Japan) is able to detect various urine contents including pathogens, RBCs, WBCs, cast and crystals, etc., and is employed in routine urinalysis.

Specifically, Atyp.C is a parameter of UF-5000 that distinguishes cells with varying nucleic acids contents, where the dye can pass through cell membrane and bind to nucleic acids. As atypical/ malignant cells tend to exhibit excessive chromatin proliferation, Atyp.C can be potentially exploited to identify them from normal cells.

In this manuscript, unstained, unfixed and uncentrifuged specimens were analyzed with UF-5000, following preparation of the UTCy slide. The results of Atyp.C assay were collected in all specimens, simultaneously. The UF-5000 analysis was completed within 2 h of sample collection. The fluorescence intensity of normal cells was assigned a baseline value of 0; cells with fluorescence intensity ≥0.1 were considered as atypical/malignant by Atyp.C assay.

### Urinary cytopathology

**Fig. 1:**
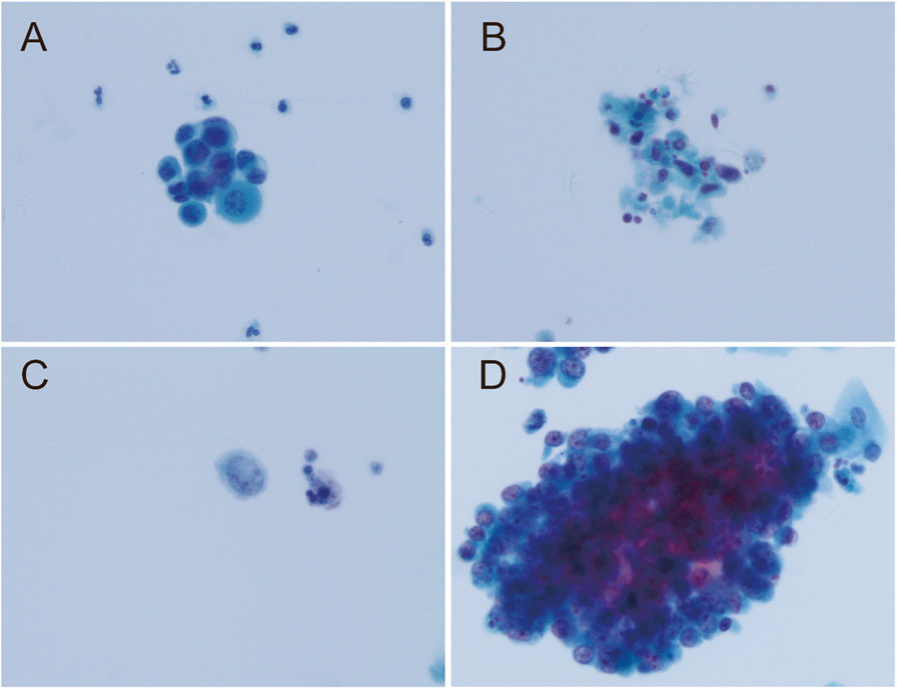
Representative images of atypical and malignant cells. **a** Atypical cells. Some cells exhibit indistinct cytoplasmic borders and an increased nucleus-to-cytoplasm ratio; histological assessment of the patient revealed LGUC. **b** Atypical cells. The cells have hyperchromatic nuclei, irregular nuclear membrane, and a high nucleus-to-cytoplasm ratio; histological assessment of the patient revealed HGUC. **c**-**d** Malignant cells. A single cell and cell cluster, respectively; histological assessment of the patient revealed HGUC (Papanicolaou stain, original magnification 400×). LGUC, low-grade urothelial carcinoma; HGUC, high-grade urothelial carcinoma

Over the study period, all UTCy slides were read and diagnosed by one of two board-certified cytopathologists, both with an average of > 10 years of experience. Consultations with specialized cytopathologists were conducted to ensure cytology consensus when necessary. The morphological features of atypical or malignant urothelial cells were defined as follows: unusual cell size and shape; irregular nuclear borders; increased nucleus-to-cytoplasm ratio; increased number of nucleoli (Fig. 1a, b) [7–9]. The cells were clustered or separate (Fig. 1c, d). In this study, cytological diagnoses were classified into the following four categories: benign/negative; atypical urothelial cells; suspicious for malignancy; and confirmed malignancy [10].

### Follow-up histological diagnoses associated with UTCy

The pathology reports of the urothelial lesions sent for further tests ordered by an urologist were retrieved from the local electronic health record system. The decision to perform further tests was based on patient preference, UTCy results, and practice pattern. Further tests performed outside our hospital were lost to follow-up.

### Statistical analysis

SPSS v17.0 (SPSS, Chicago, IL, USA) for Windows and Microsoft Excel 2010 (Redmond, WA, USA) were used for statistical analyses. Cohen’s kappa concordance coefficient was calculated to determine agreement between the methods; differences were tested using chi-squared (χ^2^) or Fisher’s exact tests, as appropriate. Correlation coefficients were categorized as slight (0.00–0.20), fair (0.20–0.40), moderate (0.40–0.60), substantial (0.60–0.80), and almost perfect (0.80–1.00) agreement. The sensitivity, specificity, and positive and negative predictive values were calculated using cross-tabulation. A *p*-value of < 0.05 was considered statistically significant.

## Results

### Patient demographics

**Fig. 2:**
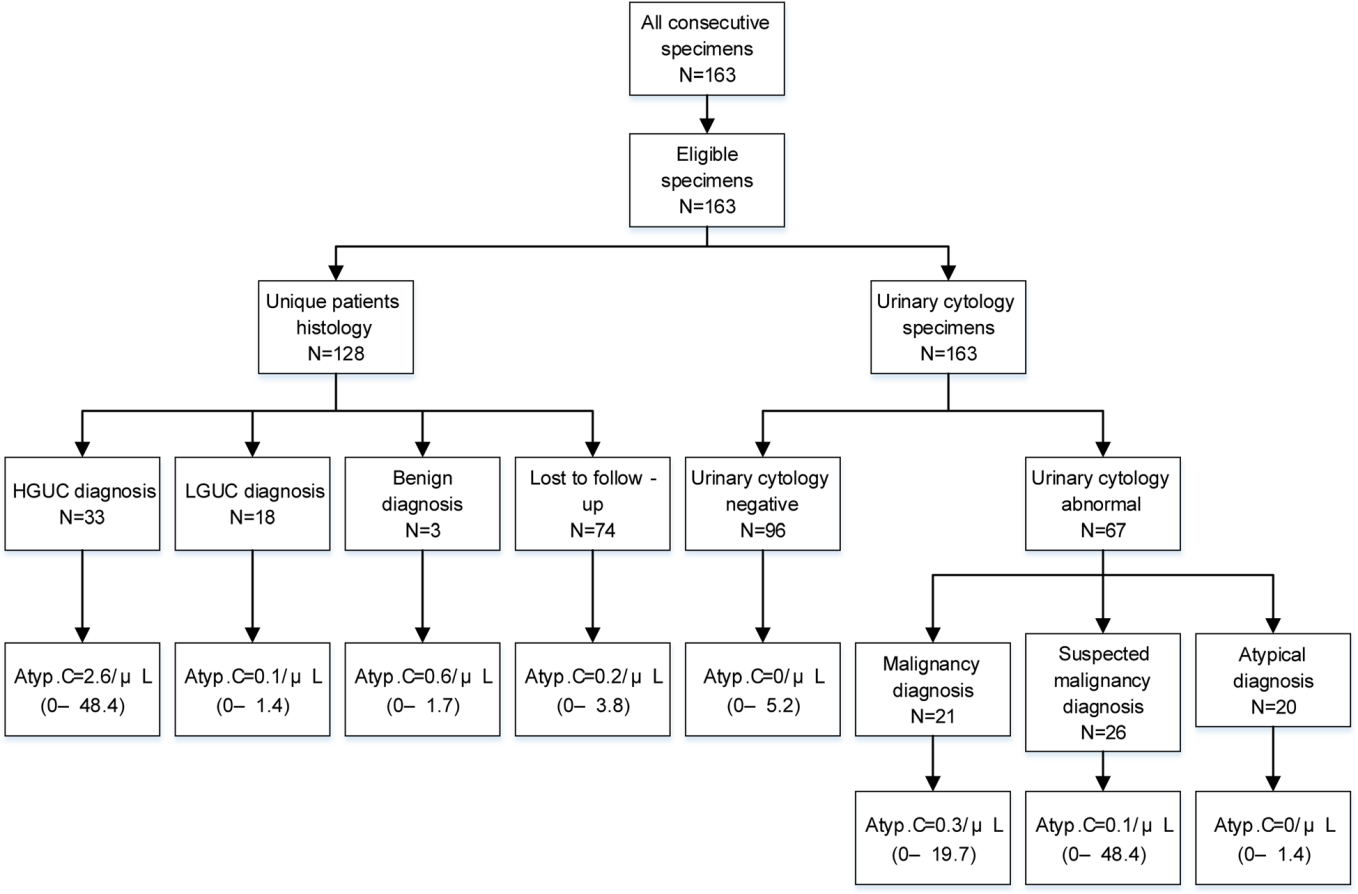
A study flow diagram according to patient demographics and the results of Atyp.C intensity

A total of 163 specimens from 128 patients were included in the study (Table 1). The median age of the patients was 64 (range, 22–101) years; 66.4% (85/128) were men and 33.6% (43/128) were women. Of these, 78.1% (100/128) were the first cytology specimens; the remaining specimens were the second or later. Of the 163 specimens, 67 (41.1, 95% CI [33.5, 48.7]) showed abnormal findings in cytology analysis. In detail, 20 specimens (12.3, 95% CI [7.2, 17.4]) were diagnosed as atypical urothelial cells, 26 specimens (16.0, 95% CI [10.3, 21.6]) as suspicious for malignancy, and 21 specimens (12.9, 95% CI [7.7, 18.1]) as confirmed malignancy. A study flow diagram is shown in Fig. 2.

**Table 1:** Patient characteristics

Characteristic	No.
No. of patients	128
No. of specimens	163
Median age (range), years	64 (22–101)
Sex	
Male	85 (66.4%)
Female	43 (33.6%)

### Comparison of UF-5000 VS cytopathology in detecting atypical urothelial cells

Table 2 shows the predictive performance of the Atyp.C assay, using UTCy results as a reference standard. Of the 20 specimens diagnosed as atypical urothelial cells in UTCy, four and 16 had positive and negative Atyp.C results, respectively. Of the 26 specimens diagnosed as suspicious for malignancy, 18 and eight had positive and negative Atyp.C results, respectively. Of the 21 specimens diagnosed as confirmed malignancy, 19 and two had positive and negative Atyp.C results, respectively. Of the remaining 96 specimens with negative diagnoses, 18 and 78 had positive and negative results, respectively. In total, 119 specimens (73.0, 95% CI [66.1, 79.9]) were correctly discriminated by Atyp.C, with a sensitivity of 61.2%, specificity of 81.3%, positive predictive value of 69.5%, negative predictive value of 75.0%, and kappa value of 0.432 ± 0.072; this agreement rate was considered “moderate.”

**Table 2:** Cross-tabulation of UTCy and Atyp. C test results

		UF-5000			Kappa	χ^2^	*P*	SEN%	SPE%	PPV%	NPV%
			Positive	Negative							
UTCy	Abnormal	AUC	4	16	0.432	30.8	< 0.001^a^	61.2	81.3	69.5	75.0
		S-malignancy	18	8							
		Malignancy	19	2							
	Negative		18	78							

*Abbreviations*: *AUC* atypical urothelial cell, *S-malignancy* suspicious malignancy, *SEN* sensitivity, *SPE* specificity, *PPV* positive predictive value, *NPV* negative predictive value

^a^Fisher’s exact test was used to compare UTCy and Atyp. C positive/negative only

The diagnosis of atypia on urinary cytopathology is a big challenge for cytopathologists [8]. Therefore, we defined “positive UTCy results” as suspicious for or confirmed malignancy. After excluding 20 specimens diagnosed as atypical cells via UTCy, a sensitivity of 81.6%, specificity of 81.1%, positive predictive value of 64.6%, negative predictive value of 91.3%, and agreement of 81.3% were obtained in all 128 patients by Atyp.C.

### Follow-up histological diagnosis associated with the UF-5000

**Fig. 3:**
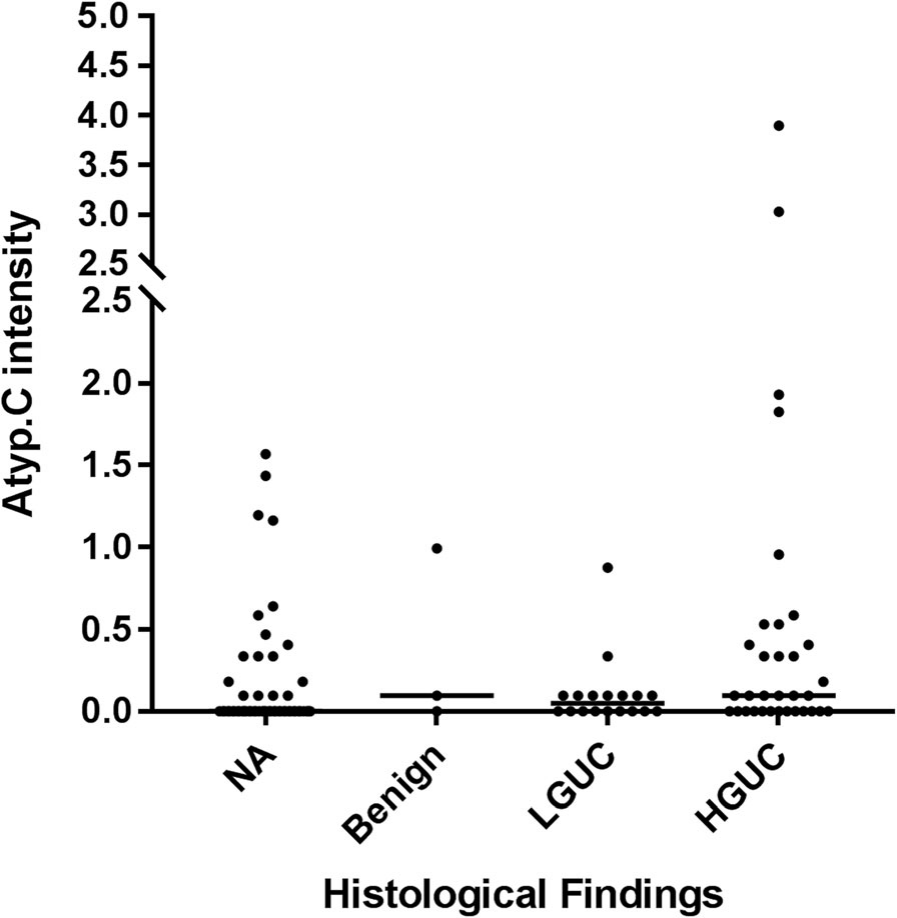
A dot plot showing Atyp.C intensity in benign, LGUC and HGUC groups identified by histology. NA, not available (lost in the follow-up); LGUC, low-grade urothelial carcinoma; HGUC, high-grade urothelial carcinoma. The original Atyp.C data was transformed as “ln (Atyp.C intensity+1)” in the Y axis

Of the 128 patients (163 specimens), 54 were further assessed histologically and 74 patients were lost to follow-up. Fifty-one histological diagnoses were classified as high- (high-grade urothelial carcinoma, HGUC) or low- (low-grade urothelial carcinoma, LGUC) grade urothelial carcinoma and the rest three were diagnosed as benign, based on the World Health Organization/International Society of Urological Pathology criteria [11]. Atyp.C level did not significantly differ between the HGUC and LGUC groups, based on histological diagnosis (Table 3). However, a dot plot revealed that a relatively large proportion of cells showed higher Atyp.C intensity in HGUC group (Fig. 3). In 51 histological positive diagnosis patients, 15 had negative results in UTCy. Of these 15 patients, two and 13 had positive and negative Atyp.C results, respectively. Of the three patients diagnosed as benign, two and one had positive and negative Atyp.C results, respectively. While using follow-up histologic diagnosis of urothelial carcinoma with or without UTCy as a reference gold standard, 91 patients (71.1, 95% CI [63.2, 78.9]) were correctly discriminated by the index test, with a sensitivity of 59.0%, specificity of 82.1%, positive predictive value of 75.0%, negative predictive value of 68.8%, and kappa value of 0.415 ± 0.079; this agreement rate was considered “moderate”, as shown in Table 4.

**Table 3:** Histological diagnosis in patients associated with the UF-5000

Histology	*n*	Atyp.C intensity median (range)	*P*
HGUC	33	2.6/μL (0–48.4)	> 0.05^*^
LGUC	18	0.1/μL (0–1.4)	
Benign	3	0.6/μL (0–1.7)	
NA	74	0.2/μL (0–3.8)	

Median of Atyp. C intensity was used due to the skewed distribution of the data

*Abbreviations*: *NA* not analyzed (not followed-up), *LGUC* low-grade urothelial carcinoma, *HGUC* high-grade urothelial carcinoma

^*^Comparing HGUC and LGUC only, Mann–Whitney *U* test, *P* = 0.110

**Table 4:** Cross-tabulation of a histologic diagnosis on follow up biopsies and Atyp. C test results

		Atyp.C		Kappa	χ^2^	*P*	SEN%	SPE%	PPV%	NPV%
		Positive	Negative							
Reference standard^a^	Positive	36	25	0.415	23.0	< 0.001	59.0	82.1	75.0	68.8
	Negative	12	55							

^a^Reference standard was histologic diagnosis with or without suspicious/malignancy on UTCy

*Abbreviations*: *SEN* sensitivity, *SPE* specificity, *PPV* positive predictive value, *NPV* negative predictive value

## Discussion

In this study, we evaluated the performance the Atyp.C parameter, determined using an automated urine analyzer (UF-5000), in patients with a suspected diagnosis of urothelial carcinoma. A recent World Health Organization/International Consultation on Urological Diseases consensus statement described an optimal new assay for the diagnosis of urothelial carcinoma as simpler, superior, more rapid, and less costly [12]. Over the past decade, many molecular assays have been evaluated and have resulted in considerable advances in the diagnosis of urothelial carcinoma, compared to urinary cytopathology; however, these are not simpler, more rapid, or less costly than the available methods [13]. In contrast, automated urine particle analysis is widely used in clinical practice [14–16]. The UF-5000 is an important device that can assess uncentrifuged specimens and rapidly provide urine particle results. The UF-5000 may meet some of the World Health Organization/International Consultation on Urological Diseases requirements for an optimal assay; moreover, no previous study has assessed the value of Atyp.C.

With urinary cytopathology as the reference standard, we demonstrated that the UF-5000 has the potential to identify abnormal cells in unstained, uncentrifuged specimens; it exhibited an agreement of 73.0%, compared to urine cytology. Of the 163 specimens examined, 67 showed abnormalities (atypical, suspicious for malignancy and confirmed malignancy) by urinary cytopathology. In our assessment of Atyp.C performance using the UF-5000, we found 16 false-negative diagnoses of atypical urothelial cells, eight false-negative diagnoses of suspicious for malignancy, and two false-negative diagnoses of malignancy. Atyp.C appeared to have much better performance in identifying malignant cells than atypical cells. The findings were similar in the re-evaluation by follow-up histologic evidence; the median and range of Atyp.C fluorescence intensity was greater in high-grade urothelial carcinoma than in low-grade urothelial carcinoma (range 0–48.4 vs. 0–1.4), although this difference was not statistically significant (*P* = 0.110). However, the dot plot revealed that a relatively large proportion of cells showed higher Atyp.C intensity in HGUC group. In this sense, higher Atyp.C intensity may be a good indicator for histological diagnosis HGUC. As is well known, the gold standard for the presence of urothelial carcinoma is histological diagnosis. Therefore, we used the follow-up histologic diagnosis with or without UTCy as a reference gold standard, and similar findings were found. Of the 25 false negative cases, 13 had negative UTCy results. The absence of abnormal findings in the urine specimens may, however, have contributed to the false negative result in our study, either using UTCy or Atyp.C method. The false positive rate (17.9%) was relatively low in our study; false positive results could be due to the presence of cytoplasmic inclusion-bearing cells, virus-infected cells, or umbrella cells since these types of cells frequently are tetraploid, aneuploidy, or show chromosomal aberrations [13, 17]. However, we did not do confirmatory testing to draw definitive conclusions in this regard. Finally, we obtained a sensitivity of 59.0%, specificity of 82.1%, positive predictive value of 75.0%, negative predictive value of 68.8%, and agreement with the reference gold standard was 71.1%. This indicates that Atyp.C needs to improve its ability in detecting abnormal UTCy findings. Furthermore, the sensitivity of cytology in the evaluation of low-grade urothelial carcinoma is highly variable, often suboptimal. The relatively low sensitivity (59.0 *and* 61.2%) found in the evaluation of the diagnostic performance of the index test could be attributed to the inclusion of atypical cases. If only malignancy or S-malignancy on UTCy were considered as a reference standard, the sensitivity was 81.6%, specificity 81.1%, positive predictive value 64.6%, negative predictive value 91.3% and the false negative cases decreased to 7. Similar findings were found using high-grade carcinoma on biopsy as the gold standard (SEN = 78.1%, NPV = 91.3%, with or without malignancy/S-malignancy on cytology). Notably, the measured fluorescence intensity of Atyp.C may be affected by the inherent differences between atypical and malignant cells such as cell membrane permeability and nucleus atypia, and this may be the explanation that sensitivity, specificity, positive and negative predictive value were improved after excluding atypical samples. Based on results of this study, the Atyp.C may have a potential role in some clinical settings, especially in screening for malignant cells in fresh urine specimens. However, the assay requires further investigation and optimization.

This study had several limitations. Apart from the single-center study design, the lack of correlation between histologic pathology and UTCy may result in, at least in part, high false-negative rate found in this study. Another inherent flaw of fluorescence flow cytometry is the presence of abnormally high nucleic acid contents in various cell types, including atypical/malignant cells, cytoplasmic inclusion-bearing cells, virus-infected cells, and umbrella cells that could have been attributed to the false positive results of UF-5000. Although Atyp.C is available, there are no clear guidelines regarding the procedure to perform on urine cytology specimens or how specimens should be preselected or preprocessed for the Atyp.C assay to ensure its clinical value.

## Conclusions

Routine urinalysis is often performed in symptomatic and asymptomatic patients. Therefore, greater consideration should be given to the presence of Atyp.C, regardless of symptoms associated with urothelial carcinoma. Based on our results, we presume that the Atyp.C assay may be useful as an accessory test in patients with a suspected diagnosis of urothelial carcinoma, or in patients requiring urothelial carcinoma surveillance, especially in identifying some high-risk patients who may need closer follow-up or additional medical treatment.

## Data Availability

Not applicable.
